# Precision Medicine Management of Chronic Lymphocytic Leukemia

**DOI:** 10.3390/cancers12030642

**Published:** 2020-03-10

**Authors:** Riccardo Moia, Andrea Patriarca, Mattia Schipani, Valentina Ferri, Chiara Favini, Sruthi Sagiraju, Wael Al Essa, Gianluca Gaidano

**Affiliations:** Division of Hematology, Department of Translational Medicine, Università del Piemonte Orientale and Azienda Ospedaliero-Universitaria Maggiore della Carità, Via Solaroli 17, 28100 Novara, Italy; riccardo.moia@uniupo.it (R.M.); andrea.patriarca@uniupo.it (A.P.); 20009611@studenti.uniupo.it (M.S.); valentina.ferri@uniupo.it (V.F.); chiara.favini@med.uniupo.it (C.F.); sruthi.sagiraju@uniupo.it (S.S.); waelmalessa123@gmail.com (W.A.E.)

**Keywords:** chronic lymphocytic leukemia, target therapy, precision medicine

## Abstract

Chronic lymphocytic leukemia (CLL) is the most common type of leukemia in western countries, with an incidence of approximately 5.1/100,000 new cases per year. Some patients may never require treatment, whereas others relapse early after front line therapeutic approaches. Recent whole genome and whole exome sequencing studies have allowed a better understanding of CLL pathogenesis and the identification of genetic lesions with potential clinical relevance. Consistently, precision medicine plays a pivotal role in the treatment algorithm of CLL, since the integration of molecular biomarkers with the clinical features of the disease may guide treatment choices. Most CLL patients present at the time of diagnosis with an early stage disease and are managed with a watch and wait strategy. For CLL patients requiring therapy, the CLL treatment armamentarium includes both chemoimmunotherapy strategies and biological drugs. The efficacy of these treatment strategies relies upon specific molecular features of the disease. *TP53* disruption (including both *TP53* mutation and 17p deletion) is the strongest predictor of chemo-refractoriness, and the assessment of *TP53* status is the first and most important decisional node in the first line treatment algorithm. The presence of *TP53* disruption mandates treatment with biological drugs that inhibit the B cell receptor or, alternatively, the B-cell lymphoma 2 (BCL2) pathway and can, at least in part, circumvent the chemorefractoriness of *TP53*-disrupted patients. Beside *TP53* disruption, the mutational status of immunoglobulin heavy variable (IGHV) genes also helps clinicians to improve treatment tailoring. In fact, patients carrying mutated IGHV genes in the absence of *TP53* disruption experience a long-lasting and durable response to chemoimmunotherapy after fludarabine, cyclophosphamide, and rituximab (FCR) treatment with a survival superimposable to that of a matched general population. In contrast, patients with unmutated IGHV genes respond poorly to chemoimmunotherapy and deserve treatment with B cell receptor inhibitors. Minimal residual disease is also emerging as a relevant biomarker with potential clinical implications. Overall, precision medicine is now a mainstay in the management and treatment stratification of CLL. The identification of novel predictive biomarkers will allow further improvements in the treatment tailoring of this leukemia.

## 1. Introduction

Chronic lymphocytic leukemia (CLL) is one of the most frequent B-cell malignancies and the most frequent leukemia in Western countries [1,2]. The extensive body of molecular studies in CLL has allowed a better understanding of the disease pathogenesis and have led to the identification of molecular biomarkers that help clinicians in the precision management of individual patients [2,3,4,5,6,7]. The identification of molecular predictors, coupled with the introduction of innovative and highly efficacious drugs in the therapeutic armamentarium, allows optimization of the treatment strategy for CLL in individual patients.

The concept of precision medicine applied to neoplastic disorders implies the individual tailoring of management and treatment of the disease on the basis of the tumor genes, coupled with host’s features. In this review, we will provide a translational perspective of the precision management of the various phases of CLL, including asymptomatic patients, patients requiring first line therapy, relapsed/refractory (R/R) disease, and Richter syndrome (RS).

## 2. Genomics and Biology of CLL as the Backbone for a Precision Medicine Approach

**Figure 1:**
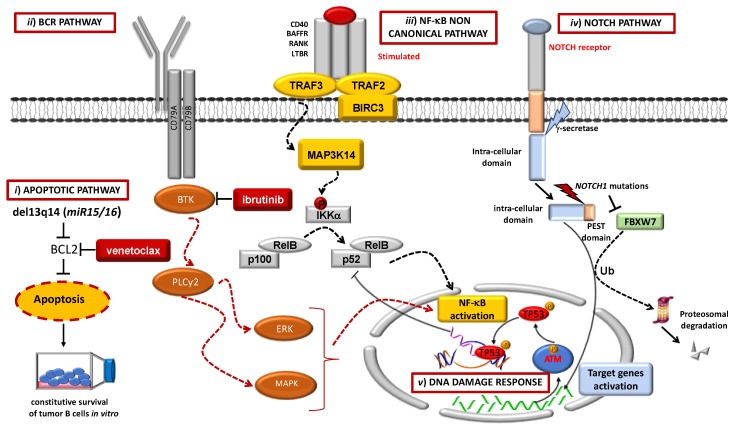
Biological pathways involved in chronic lymphocytic leukemia (CLL) pathogenesis and harboring therapeutic implications. (i) Venetoclax (in red) binds and inhibits the BCL2 anti-apoptotic protein, which is frequently impaired by a variety of molecular mechanisms (e.g., del13q14), thus restoring the apoptosis in CLL cells. (ii) Bruton’s tyrosine kinase (BTK), a pivotal kinase located downstream to the BCR pathway, can be targeted by BTK inhibitors such as ibrutinib (in red). (iii) The activation of the non-canonical NF-κB pathway contributes to cell survival and progression. (iv) Notch homolog 1 (NOTCH1) mutations disrupt the PEST domain (in orange) of the NOTCH intracellular domain (NICD), leading to constitutive transcription of target genes promoting survival and proliferation. (v) The nuclear TP53 and Ataxia telangiectasia mutated (ATM) proteins are involved in the DNA damage response pathway.

An extensive body of molecular studies has deciphered the molecular landscape of CLL [2,3,4,5,6,7]. CLL is not characterized by a unique and unifying genetic lesion, but rather displays a variety of molecular abnormalities that are responsible for disease pathogenesis, progression, and transformation. Different biological pathways are involved in CLL pathogenesis and are deregulated by different genetic lesions [2]. Figure 1 shows the main biological pathways involved in CLL pathogenesis and harboring therapeutic implications.

As for all B cell malignancies, the unique immunoglobulin heavy variable (IGHV) gene rearrangement is the hallmark of every single CLL clone. Pivotal studies have demonstrated that the IGHV gene repertoire in CLL is skewed, implying a role for antigen selection in disease development [8,9]. Another important feature of IGHV genes utilized by CLL is the degree of identity of the IGHV rearrangement to the normal counterpart. In approximately 60% CLL, the IGHV genes utilized by the leukemic clone display a homology to the normal counterpart of less than 98%. These cases are termed IGHV-mutated CLL and are postulated to originate from B cells that have undergone somatic hypermutation of immunoglobulin genes, a physiological phenomenon of B cell transit through the germinal center. Conversely, 40% of CLLs display IGHV genes with a homology to the normal counterpart equal to or higher than 98%. These cases are termed IGHV-unmutated CLL and are postulated to derive from naïve B cells that have undergone maturation independent of the germinal center reaction. The mutational status of IGHV genes identifies CLL subgroups that differ significantly, both molecularly and clinically. IGHV-unmutated CLL is associated with adverse prognostic genomic aberrations, increased B cell receptor signaling (BCR) capacity, shorter time to progression, and inferior survival compared to IGHV-mutated patients [10,11,12]. Beside its prognostic value, the mutational status of IGHV genes also represents a predictive biomarker, since CLL patients with mutated IGHV genes and devoid of *TP53* abnormalities may still benefit from chemoimmunotherapy (CIT), which is otherwise considered a suboptimal treatment for IGHV-unmutated patients [13,14,15].

The apoptosis pathway is frequently impaired and the most frequent genetic alteration of CLL, namely deletion of 13q14 (del13q14), is a key feature for apoptosis deregulation in many, though not all, CLL patients. Del13q14 is present in 50% to 60% cases and more frequently occurs as a monoallelic lesion [16]. Del13q14 is an early event in CLL pathogenesis and may be present already at the stage of monoclonal B-lymphocytosis (MBL), which frequently precedes CLL diagnosis [17]. The minimal deleted region on 13q14 encompasses two micro RNA (miR), namely miR-15 and miR-16, that physiologically inhibit the function of the anti-apoptotic protein BCL2 [18]. Loss of miR-15/16 removes an inhibitor of BCL2 expression, and therefore promotes the constitutive survival of tumor B cells in vitro and leads to CLL development in mouse models [19,20]. Patients with del13q14 are characterized by a good prognosis if they do not concomitantly harbor other genetic lesions (e.g., 17p deletion, 11q deletion) that are associated with a poorer outcome [16]. Although del13q14 has unraveled the relevance of the apoptotic pathway in the disease and its assessment by Fluorescent In Situ Hybridization (FISH) is recommended by guidelines [21], this genetic alteration is not currently used as a biomarker for precision medicine, since CLL responds to BCL2 inhibitors independent of del13q14 status.

A pivotal pathway involved in CLL pathogenesis and markedly affecting treatment response is the DNA damage response pathway [2]. The most frequent lesions of genes belonging to this pathway are molecular alterations of *TP53* and *ATM* [22]. The *TP53* gene codes for a central regulator of the DNA damage-response pathway and is the target of the genotoxic effect of chemotherapy. Chemotherapy acts by inducing DNA damage, thus activating the *TP53* pathway, which leads to apoptosis of CLL cells. Conversely, when *TP53* is disrupted by mutation and/or deletion, chemotherapy fails to induce apoptosis in CLL cells, that, consequently, may proliferate at a sustained pace and accumulate multiple additional genetic lesions that promote progression and clonal evolution [23]. Consistently, CLL patients with *TP53* disruption have a very poor response to CIT and are candidates for treatment with new drugs [13,21,24]. *ATM* is a tumor suppressor gene that is crucial for the DNA damage response. *ATM* is located in the 11q22-23 region, which is deleted in approximately 15–20% of newly diagnosed CLL cases. Patients with del11q or *ATM* mutations are associated with an intermediate prognosis [22]. 

The nuclear factor-κB (NF-κB) signaling pathway is a key component of CLL pathogenesis and encompasses two pathways, termed canonical and non-canonical [25]. The canonical pathway is enhanced by BCR signaling, whereas the non-canonical pathway is usually activated by cytokines or by other microenvironmental interactions [26]. In CLL, *BIRC3*, a negative regulator of non-canonical NF-κB, is frequently disrupted, leading to aberrant and constitutive activation of this biological pathway, promoting proliferation and survival [2]. *BIRC3* mutations are absent in MBL, are rare at the time of CLL diagnosis (3–4%), but are detectable in approximately 25% of fludarabine refractory patients [27]. A recent study has demonstrated that *BIRC3* mutations mediate chemorefractoriness to fludarabine, cyclophosphamide, and rituximab (FCR), both in vitro and in vivo [28]. Consistently, patients with *BIRC3* mutations treated with FCR have the same poor outcome as patients with *TP53* disruption, which represents the strongest predictor of chemorefractoriness in CLL [28]. The potential value of *BIRC3* mutations as a predictor of failure after CIT is corroborated by observations from the CLL14 phase 3 clinical trial comparing chlorambucil-obinutuzumab with venetoclax-obinutuzumab in patients with previously untreated CLL [29]. In fact, *BIRC3* mutations were associated with a shorter progression-free survival (PFS) in the chlorambucil-obinutuzumab arm, reinforcing the role of *BIRC3* mutations as a biomarker of chemorefractoriness [29].

Other genetic lesions involved in CLL pathogenesis are represented by the deregulation of the NOTCH signaling pathway associated with *NOTCH1* or *FBXW7* mutations [22,30]. The *NOTCH1* gene codes for a transmembrane receptor that, upon ligand binding and migration of the NOTCH1 intracellular domain to the nucleus, induces the transcription of pro-survival and anti-apoptotic genes [31]. *NOTCH1* mutations usually occur within the PEST domain, which harbors the aminoacidic sequences recognized by the ubiquitin ligase F-box and WD repeat-containing protein 7 (*FBXW7*). Physiologically, the FBXW7 protein recognizes the PEST domain of the NOTCH1 protein, and, upon ubiquitination, induces its degradation through the proteasomal pathway. Consistently, in the case of *NOCTH1* mutations that disrupt the recognition sequence in the PEST domain, the NOTCH1 protein does not undergo proteasomal degradation, and rather retains its function as a positive transcription factor for NOTCH1 target genes [22]. NOTCH1 signaling may also be enhanced by mutations of *FBXW7* that impair the ubiquitination of the NOTCH1 protein [32,33]. Mutations of the *NOTCH1* gene are present in approximately 10% of CLL patients at diagnosis and are increased in R/R patients [34]. Although *NOTCH1*-mutated patients have a shorter survival compared to wild type patients, the mutational screening of *NOTCH1* has not entered the clinical practice until now due to the lack of conclusive evidence that *NOTCH1* mutations are a solid predictor for treatment choices [34]. Assessment of the clinical value of *NOTCH1* mutations in the CLL8 trial, comparing FCR with FC in first line CLL therapy, has demonstrated that *NOTCH1*-mutated patients may not benefit from the addition of rituximab to the FC backbone [24]. Consistently, CLL cells from *NOTCH1*-mutated cases are characterized by lower CD20 expression and by a lower extent of cell lysis induced by anti-CD20 exposure in vitro compared to *NOTCH1* wild type patients. Also, CD20 expression on CLL cells is upregulated by the blockade of NOCTH1 signaling exerted by γ-secretase inhibitors or NOTCH1-specific small interfering RNAs [35]. These biological findings possibly reflect a deregulated epigenetic loop associated with the impaired function of histone deacetylases (HDAC) that is induced by *NOTCH1* mutations and is partially restored by treatment with HDAC inhibitors [35]. The novel anti-CD20 antibody obinutuzumab, provided with a higher efficacy compared to rituximab, has been shown to overcome the refractoriness to anti-CD20 therapy in CLL carrying mutations of *NOTCH1* [36]. *NOTCH1* mutations frequently co-occur with trisomy 12, a genetic lesion found in approximately 15% of CLL patients at the time of diagnosis [22]. Patients with trisomy 12 are considered a group with an intermediate prognosis and have a higher risk of RS transformation [22]. Despite its recurrence and prognostic importance, the mechanisms by which trisomy 12 contributes to CLL pathogenesis are still unknown.

Splicing is another recurrent molecular process that is deregulated in CLL [2,22]. The most frequent gene mutations involved in this pathway target the *SF3B1* gene [37]. This gene codes for a fundamental part of the U2 small nuclear ribonucleoprotein (snRNP) essential for the initial phases of RNA splicing. The consequences of *SF3B1* mutations are not completely understood but seem to generate aberrant splicing of genes coding for proteins involved in different biological pathways, including DNA damage response [22]. *SF3B1* is mutated in approximately 10% of newly diagnosed CLL patients and is associated with a worse outcome than wild type cases [37]. Recently, the U1 spliceosomal RNA gene has been described to be somatically mutated in different types of cancers, including CLL [7]. From a biological standpoint, this mutation creates novel splice junctions and alters the splicing pattern of multiple genes. This mutation is present in approximately 3–4% of CLLs at diagnosis and is associated with a shorter time to first treatment (TTFT) [7]. Currently, mutations of spliceosome genes do not yet affect management or treatment decisions in CLL.

## 3. Assessment of Biological Prognosticators and Predictors

A prognosticator is a clinical or biological feature that provides information about the natural history and the prognosis of the disease independent of the treatment received [38]. Conversely, a predictor is a biomarker that provides information on the likely benefit from a specific treatment [38]. Among CLL genetic lesions, *TP53* abnormalities and IGHV mutational status currently fulfill the criteria of predictive biomarkers whose usage is recommended by guidelines for the clinical management of CLL [21,39].

As mentioned above, CLL patients with *TP53* disruption, by either deletion or mutation, are refractory (i.e., failing treatment or progressing within six months from treatment start) to CIT regimens [2,21]. The introduction of biological drugs that inhibit the BCR pathway or inhibit BCL2 have mitigated, though not completely abolished, the negative impact of *TP53* disruption. On these grounds, patients with *TP53* disruption are treated upfront with biological drugs whose mode of action is independent of the DNA damage response [21]. Conversely, patients with mutated IGHV genes devoid of *TP53* disruption may still benefit from CIT, and until now, phase 3 clinical trials comparing CIT to biological drugs have not demonstrated the superiority of biological drugs compared to CIT in this subgroup of patients [38,40,41].

According to guidelines, *TP53* status must be assessed by FISH and by mutational analysis before starting treatment and at every subsequent relapse [21]. The mutational status of IGHV genes must also be tested before starting treatment, but, since its pattern does not change over time, it does not need to be retested at the time of relapse [21]. At the time of diagnosis in the absence of treatment indications, testing for *TP53* abnormalities or for IGHV mutational status should not be performed in the clinical practice and is restricted to research purposes. Importantly, indications for starting treatment do not depend on the results of these tests but only on the patient’s clinical stage and symptoms [21].

Since *TP53* and IGHV mutational status guides treatment choices, the analysis of these two molecular predictors needs to be harmonized across laboratories using validated methodologies and guidelines [42,43]. The European Research Initiative on CLL (ERIC) has generated guidelines for the analysis of both *TP53* and IGHV mutational status and provides the possibility of an accreditation process that is being offered worldwide to centers [42,43]. Peripheral blood is an appropriate material for *TP53* mutation analysis when lymphocyte count is >10 × 10^9^/L [42]. The sequenced region of the *TP53* gene must include exons 4–10, including the DNA-binding domain and the oligomerization domain. Optimally, exons 2, 3, and 11 should also be analyzed to cover the entire coding region [42]. The *TP53* gene may be sequenced by Sanger sequencing or by Next Generation Sequencing (NGS) using the cut of 10% of variant allele frequency for variant calling, since the clinical impact of small subclones of *TP53* is not yet completely understood [42]. A few studies have demonstrated that *TP53* mutations with a variant allele frequency below the conventional 10% threshold are also associated with a worse outcome in patients treated with CIT [44,45]. However, these initial studies are retrospective in nature, and further evidence from prospective trials should be acquired before the clinical value of small *TP53* mutated subclones might be reassessed for treatment decisions.

Regarding IGHV genes, peripheral blood is an appropriate material for the test, and purification of B cells is usually not necessary unless the patient presents with a low fraction of leukemic cells [43]. According to the ERIC guidelines, leader primers are the first choice, since they allow the amplification of the entire sequence of the rearranged IGHV gene. By this approach, the true and complete level of somatic hypermutation of IGHV genes utilized by the CLL clone can be determined [43]. After sequencing, specific bioinformatic tools allow the analysis of the IGHV rearrangement [43].

The ERIC network has generated large datasets that allow the analysis of thousands of molecular IGHV sequencing data [46,47]. A fraction of unrelated CLL patients carry quasi-similar, if not identical, IGHV sequences, termed stereotyped BCR [46,47]. Different groups of stereotyped BCR sequences have been identified, some of which are characterized by unique molecular and clinical features [46,47]. For instance, the stereotyped BCR subset #2 identifies a subgroup of CLL patients who, despite carrying mutated IGHV genes, are characterized by a very poor outcome and may deserve novel therapeutic strategies [48]. The inclusion of stereotyped BCR subsets among predictive biomarkers is an attractive possibility for a precision medicine approach to CLL in the future. Current guidelines, however, do not include stereotyped BCR subsets among the biomarkers used for choosing treatment in the clinical practice.

Minimal residual disease (MRD) assessment has become an important endpoint in clinical trials and is being considered by the European Medicines Agency as an endpoint in clinical trials [49]. MRD assessment is recommended in clinical trials using standardized protocols of either four-color flow cytometry or allele-specific oligonucleotide PCR (with a sensitivity of 10^−4^) [49]. MRD has emerged as a strong predictor of outcome both in patients treated with CIT and in patients treated with biological drugs and may become a potential tool to decide the timing of drug interruption [49]. Currently, however, assessment of MRD is not recommended by guidelines as a clinical test for the clinical practice of CLL [21].

## 4. Management of Asymptomatic CLL Patients

In most cases, CLL is an incidental diagnosis, discovered after a complete blood count performed for other reasons [49]. Moreover, 70% of newly diagnosed CLL patients present in an early stage, according to the Binet and Rai staging systems, may never require treatment, and may have a life expectancy similar to the general population [50,51]. Despite the indolent behavior of CLL in the majority of cases, some patients have a CLL clone with a high proliferation rate that may lead to early treatment requirement due to progressive lymphocytosis, enlarged lymph nodes, cytopenia, and systemic symptoms [21].

Asymptomatic early stage CLL patients are currently managed with a watch and wait strategy, and treatment is started only in cases of symptomatic disease, according to the latest International Workshop on Chronic Lymphocytic Leukemia (iwCLL) guidelines [21]. Two clinical trials comparing chlorambucil and fludarabine versus placebo in asymptomatic CLL patients did not demonstrate an advantage in survival of early treatment versus observation [52,53]. Preliminary results of the CLL12 clinical trial, a phase 3 trial comparing ibrutinb with observation in asymptomatic CLL, demonstrate a higher PFS in the ibrutinib arm, but results are not considered mature enough to demonstrate an advantage of ibrutinib versus observation in terms of overall survival [54]. Therefore, early intervention in CLL without clinical indications for treatment is not currently justified, and guidelines recommend a watch and wait strategy for these patients [21]. Molecular analysis of the CLL12 trial may reveal whether patients with specific genetic lesions may potentially benefit from early treatment, prompting the future design of clinical trials aimed at assessing the value of intervention for early stage CLL patients with high risk molecular features. Clinical trials for asymptomatic early stage CLL patients are reported in Table 1.

Recent studies have tried to identify the clinical and molecular features of early stage CLL patients managed with a watch and wait approach and who might manifest treatment requirement soon after diagnosis [6,55,56,57,58]. The pattern of tumor growth of untreated CLL has been investigated by analyzing serial longitudinal samples collected between diagnosis and the time of treatment requirement [6]. Two different patterns of growth have been identified. The exponential growth pattern is characterized by a rapid proliferation of the CLL clone, whereas the logistic growth pattern displays a lower rate of progression [6]. These two different patterns of growth associate with peculiar molecular features. CLL cases with an exponential growth are mainly IGHV-unmutated CLL and have a higher frequency of clonal and subclonal somatic genetic lesions compared to patients with a logistic growth pattern [6]. These different growth patterns, as well as the association between exponential growth, unmutated IGHV genes, and additional genetic lesions, have been validated in an independent cohort of CLL patients [6]. 

Other studies have focused on the identification of clinical and molecular features that might identify early stage CLLs who are at risk of early progression at the time of diagnosis. Such patients, if identified a priori, might benefit from clinical trials comparing early intervention versus observation. In this context, the combination of simple clinical features and molecular biomarkers, namely lymphocyte count > 15,000/µL, palpable lymph nodes, and unmutated IGHV genes, identifies three different subgroups of Binet A and treatment naïve CLL patients with a high risk of early treatment requirement [55]. This risk model, termed IPS-E (International Prognostic Score—Early), has been validated in several independent series and is a robust tool to inform at the time of diagnosis about the probability that a given CLL patient in early stage disease progresses and needs treatment [55].

In addition, by taking advantage of the genetic heterogeneity of CLL, mutations of genes involved in CLL pathogenesis have been tested as biomarkers for identifying early stage CLL patients with a higher risk of progression and treatment requirement. These studies point to mutations of *SF3B1*, *NOTCH1*, *ATM*, *U1*, and *XPO1* as molecular predictors of shorter TTFT [7,56,57]. Interestingly, *TP53* disruption is not associated with a shorter TTFT, in line with the notion that *TP53* disruption interacts with treatment with chemotherapeutic agents, but not with a watch and wait strategy that does not expose CLL cells carrying *TP53* disruption to the positive selection pressure exerted by ineffective chemotherapy [55,56]. The precise role of gene mutations in sorting asymptomatic CLL patients with an imminent risk of treatment requirement still needs to be clarified and is the current subject of investigations.

## 5. Precision Management of First Line Therapy in CLL

The choice of first line treatment for CLL is based on the molecular features of the disease, as well as on patient features and access to novel drugs in different geographic areas of the world. The most recent guidelines recommend testing for IGHV mutational analysis, FISH cytogenetics including 13q, 11q, and 17p deletion and trisomy12, and *TP53* mutational status before starting treatment. Patients with *TP53* abnormalities, including 17p deletion and/or *TP53* mutation, should be treated with biological drugs, avoiding CIT [21].

Recently, evidence from several phase 3 clinical trials comparing CIT versus chemo-free regimens has demonstrated the superiority of chemo-free regimens in the first line treatment of CLL. However, subgroup analysis based on IGHV mutation status has revealed significant differences between IGHV-mutated and IGHV-unmutated patients, thus reinforcing the relevance of biomarkers for a precision medicine approach to CLL patients requiring first line therapy (Table 2) [38,40,41,60].

Patients with unmutated IGHV genes demonstrated a poorer outcome when treated with CIT in all the above-mentioned trials, mandating therapy with biological agents in this molecular subgroup of patients [38,40,41,60]. Conversely, patients with mutated IGHV genes demonstrated a favorable outcome when treated with CIT, irrespective of age and of comorbidities. In the E1912 trial, designed to compare ibrutinib-rituximab with FCR in the treatment of naïve, young, and fit patients, the outcome of patients with mutated IGHV genes was superimposable in both arms [38]. Similar results have been obtained also in the phase 3 trial comparing first line bendamustine-rituximab (BR), ibrutinib, or ibrutinib-rituximab (IR) in patients ≥65 years of age, and in patients enrolled in the CLL14 trial comparing obinutuzumab-venetoclax to obinutuzumab-clorambucil in elderly patients or in patients with comorbidities [40,41]. Overall, these results document that IGHV-mutated CLL devoid of *TP53* disruption may benefit from both CIT and biological drugs, without statistical differences. At variance, in the Illuminate trial that randomized patients to receive ibrutinib-obinutuzumab or chlorambucil- obinutuzumab, the chemofree arm was superior also in the subset of mutated IGHV patients [60].

As expected, patients with *TP53* abnormalities treated in the CIT arms failed early, whereas *TP53* abnormalities did not impact patients treated with biological agents, expect for the CLL14 trials, in which *TP53*-mutated patients were also associated with a poor outcome in the obinutuzumab-venetoclax arm [29].

Several ongoing clinical trials are comparing different chemo-free regimen front lines with the aim of eradicating the CLL clone with a fixed duration therapy scheme (Table 2). A phase 2 trial in high-risk patients (harboring ≥1 of the following features: 17p deletion, *TP53* mutation, 11q deletion, unmutated IGHV) combined ibrutinib and venetoclax for 24 cycles [65]. If MRD negativity was achieved, therapy was stopped. In this high-risk population, after 12 cycles of combination therapy with ibrutinib and venetoclax, 88% of patients had complete remission, and 61% had remission with undetectable cytofluorimetric MRD [65]. These results demonstrate the synergistic action of ibrutinib and venetoclax and prompt the design of clinical trials aimed at defining the best combination for the potential eradication of the CLL clone in individual patients.

## 6. Precision Management of Relapsed/Refractory CLL Patients

The definition of CLL relapse encompasses disease progression in a patient who has previously achieved the above criteria of a complete or partial remission for ≥6 months, whereas patients failing treatment or progressing within 6 months from treatment are considered refractory [21]. Until recently, few valid therapeutic options were available for CLL patients who relapsed early after first-line CIT or were refractory to it (Table 2). The frequency of *TP53* disruption, a solid biomarker of chemorefractoriness, is in fact high in R/R CLL patients. At the time of relapse, the status of *TP53* disruption should be reassessed by FISH and mutation analysis, in particular, in cases who had scored negatively in previous phases of the disease. The mutation status of IGHV genes should not be retested, since it does not change over time during different phases of the disease.

BCR inhibitors were the first biological drugs offering a change in the natural history R/R patients with CLL. In a phase 2 clinical trial of ibrutinib with more than five years of follow up, median PFS in R/R patients was 51 months, a result that had never been achieved before [61]. Del17p, del11q, and complex karyotype (i.e., >3 chromosomal independent abnormalities) sorted out as biomarkers of shorter PFS [61]. Conversely, IGHV mutational status did not impact on PFS and the outcome of IGHV-mutated and -unmutated patients was superimposable. 

A recent update of the resonate clinical trial, a phase 3 study comparing ibrutinib and ofatumumab in R/R CLL patients, demonstrated a sustained efficacy of ibrutinib [62]. After a median follow up of 44 months, the median PFS was not reached for the ibrutinib arm, while PFS was 8.1 months for the ofatumumab arm. Even though most patients in the ofatumumab arm have crossed over to ibrutinib, the overall survival (OS) censored for crossover was significantly higher in patients randomized to ibrutinib. Subgroup analysis has demonstrated that patients with ≤ two prior lines of therapy have a better outcome than patients who receive more than two lines of prior therapy, pointing to the need for not delaying ibrutinib administration in R/R patients [62]. In patients treated in the ibrutinib arm, del 17p del, del 11q del, complex karyotype, or unmutated IGHV did not impact on PFS. A trend toward a shorter PFS, albeit not statistically significant, was found in patients with *TP53* and *SF3B1* mutations [62].

The genetic lesions described in the above paragraphs lead to reduced efficacy of ibrutinib with a molecular mechanism that is independent of the ibrutinib mode of action. Ibrutinib binds to a cysteine residue positioned at codon 481 of the BTK gene and inhibits the catalytic site of BTK. Mutations lead to a cysteine-to-serine amino acid change at codon 481 and predispose to ibrutinib resistance by altering drug binding to BTK [66]. These mutations are absent in ibrutinib-naïve CLL, and may be selected upon drug exposure [67]. An alternative mechanism of resistance to ibrutinib is represented by the constitutive activation of proteins located downstream to BTK. Consistently, gain-of-function mutations of the PLCγ2 gene in ibrutinib-resistant CLL lead to autonomous BCR activity [66,68,69]. Although BTK or PLCγ2 mutations are detected in approximately 85% of patients who progress under ibrutinb, regular monitoring of BTK or PLCγ2 mutations is not recommended by current guidelines [21,66,67,68,69]. Interestingly, novel non-covalent BTK inhibitors may overcome these detrimental genetic lesions [70].

The identification of impaired apoptosis in CLL fostered the discovery of high-affinity ligands that inhibit the anti-apoptotic BCL2 protein [71]. Navitoclax, the first anti-BCL2 small molecule, demonstrated high efficacy in R/R CLL patients but its use was limited by severe thrombocytopenia [72]. The adverse event of navitoclax is caused by the inhibition of BCLX_L_, which is highly expressed in platelets [73]. To overcome this hurdle, an orally bioavailable and BCL2-selective inhibitor termed venetoclax was developed [74,75]. The pivotal phase 1 and phase 2 studies documented the high efficacy of venetoclax in R/R CLL, with an overall response rate (ORR) exceeding 75% also in molecularly high-risk patients harboring del17p [63,76]. Based on this initial evidence, a phase 3 study compared the combination of venetoclax with rituximab (VR) and BR in R/R CLL patients [64]. A fixed duration of two years of venetoclax treatment was an innovative feature of the study. In this trial, 389 patients were randomized to receive venetoclax for up to two years, plus rituximab for the first six months (VR group) or bendamustine plus rituximab for six months (BR group). VR significantly prolonged PFS compared to BR, with a two years PFS of 84.9% for VR compared to 36.3% for BR [64]. The benefit of VR was maintained across all clinical and biologic subgroups, including patients with del17p. VR also significantly prolonged overall survival [64].

VR improved MRD negativity compared to BR. At the nine months timepoint, 62.4% of patients in the VR were MRD negative in the peripheral blood compared to 13.3% in the BR arm [64]. From the standpoint of precision medicine, it is remarkable that MRD status at the end of treatment is a strong indicator of the risk of disease recurrence; more precisely, 78.6% of patient with MRD level >10^−2^ progressed, compared to 2% of patients with MRD level <10^−4^ [77]. Future studies need to address whether patients with a high MRD load might benefit from a different management strategy that includes continuing venetoclax after the fixed duration schedule of the VR combination [77].

## 7. Precision Medicine in the Context of Richter Syndrome (RS)

RS is defined as the occurrence of an aggressive B-cell lymphoma in patients with a previous or concomitant diagnosis of CLL. Two pathological variants of RS exist, namely diffuse large B cell lymphoma (DLBCL) in 90–95% of cases and Hodgkin lymphoma in the remaining 5–10% of cases [2,78,79]. Different genetic lesions have been identified in the pathogenesis of RS transformation. These lesions involve *TP53*, *NOTCH1*, *MYC*, and *CDKN2A* abnormalities, predisposing to reduced apoptosis and uncontrolled cellular proliferation. The high frequency of *TP53* disruption in RS (up to 60–70% of cases) explains the marked chemorefractoriness of RS to standard regimens, such as (rituximab, cyclophosphamide, doxorubicin, vincristine, prednisone) R-CHOP, commonly used in this disease for induction treatment [80,81,82,83].

Beside the occurrence of molecular alterations of proto-oncogenes and tumor suppressor genes, the features of the IGHV rearrangement of the CLL clone are also important for RS development. Notably, CLL patients carrying a specific stereotyped immunoglobulin gene in the subset 8 configuration (IGHV4-39/IGHD6-13/IGHJ5) are at a very high risk of RS development, and stereotyped subset 8 BCR configuration is highly enriched in DLBCL RS, supporting a role of BCR signaling in transformation [84]. These notions imply that detecting a stereotyped subset 8 BCR configuration in CLL should raise the awareness of the clinician for potential development of RS during the subsequent clinical course [84]. In addition to the molecular features of the disease, a recent report has demonstrated the relevance of total metabolic tumor volume (TMTV) as a novel prognosticator for RS. In particular, a high pre-treatment TMTV, measured using ^18^-fluorodeoxyglucose positron emission tomography (PET), is a predictor of shorter survival in patients with RS [85].

Once the diagnosis RS is established, it is important to evaluate the clonal relationship of the RS clone with the pre-existent CLL clone [78,79,80]. The assessment of the clonal relationship between CLL and RS can be performed by comparing the IGHV rearrangement utilized by the CLL phase and by the RS phase. This analysis allows the identification of two different groups of patients with a different risk of progression and death. Patients with clonally-related RS, i.e., patients in which the CLL clone and the RS clone carry the same IGHV gene rearrangement, have a very dismal prognosis with the sole induction based on CIT [78,79]. Consistently, clonally related RS patients who are transplant-eligible are usually consolidated with allogeneic hematopoietic stem cell transplantation after induction treatment with CIT. Autologous hematopoietic stem cell transplantation might be an alternative if a donor is not available. Conversely, clonally-unrelated RS represent a secondary DLBCL arising de novo in the context of a condition, CLL, that predisposes per se to second malignancies. Clonally-unrelated RS are characterized by a better outcome and may benefit from R-CHOP without further treatment [78,79]. While the frequency of *TP53* disruption is very high in clonally-related RS, it does not exceed 20% in clonally-unrelated RS [78,79]. From a practical standpoint, the assessment of a clonal relationship between CLL and RS requires the availability of biological material (either fresh frozen or formalin-fixed paraffin embedded) from both phases of the disease and is of particular relevance in patients with a histological diagnosis of RS who are transplant eligible [78,79].

## 8. Conclusions and Perspectives

The clinical management and therapeutic landscape of CLL have changed drastically over the last few years. The availability of a variety of treatment options, ranging from CIT to molecular inhibitors of the BCR and BCL2 pathways, has raised the need for a more refined choice of the most suitable treatment strategy for each individual patient. In parallel, detailed knowledge of the CLL genome has favored the identification of biomarkers that serve as solid treatment predictors and have fostered the application of precision medicine to the clinical practice of CLL.

The current algorithm of CLL management, however, might benefit in the future from further knowledge generated by precision medicine studies. The true value of MRD as a biomarker guiding treatment duration still needs to be explored and conclusively assessed. Given the variety of biological medicines for CLL, the availability of molecular predictors helping choose among the different options would be desirable. Specific stereotyped BCR subsets are emerging as novel molecular predictors, and not only prognosticators, of high-risk CLL and treatment failure, and may potentially provide valuable biomarkers in the clinical practice. Also, molecular investigations might help identify a priori those early stage CLL that are at imminent risk of progression and treatment requirement.

## Figures and Tables

**Table 1:** Clinical trials in asymptomatic CLL patients.

Trial	Trial Status	Phase	Setting	Interventions	N. of patients	PFS	OS
Ibrutinib versus placebo in patients with asymptomatic, treatment-naïve early stage Chronic Lymphocytic Leukemia (CLL): primary endpoint results of the phase 3 double-blind randomized CLL12 trial [54]	Active, not recruiting	3	Untreated patients with stage A CLL with intermediate, high or very high risk of progression	Ibrutinib	182	Median PFS: not reached at median observation time of 31 months	-
				Placebo	181	Median PFS: 14.8 months	-
Fludarabine or Observation in Treating Patients With Stage 0, Stage I, or Stage II Chronic Lymphocytic Leukemia [53]	Completed	3	Untreated patients with stage A CLL aged 18 years or older	High Risk Patients - Fludarabine	93	Median PFS: 30.1 months	Median OS: 126.8 months
				High Risk Patients - Watch & Wait	96	Median PFS: 12.9 months	Median OS: not reached at median observation time of 8.5 years
Chlorambucil in Indolent Chronic Lymphocytic Leukemia [52]	Completed	3	Untreated patients with stage A CLL	No treatment	308	-	5 years: 80% 10 years: 54%
				Chlorambucil (0.1 mg/Kg/die)	301	-	5 years: 76% 10 years: 47%
Rituximab, Fludarabine, and Cyclophosphamide or Observation Alone in Treating Patients With Stage 0, Stage I, or Stage II Chronic Lymphocytic Leukemia [59]	Completed	3	Untreated patients with stage A CLL aged 18 years or older	High Risk Patients- FCR	100	Median PFS: not reached at median observation time of 55.6 months 5 years PFS: 55.2%	5 years: 82.9%
				High Risk Patients- Watch & Wait	101	Median PFS: 18.6 5 years PFS: 12.6%	5 years: 79.9%
				Low Risk Patients - Watch & Wait	599	Median PFS: 84.3 5 years PFS: 77.1%	5 years: 97.2%
Alemtuzumab and Rituximab in Treating Patients With High-Risk, Early-Stage Chronic Lymphocytic Leukemia [NCT00436904]	Completed	2	Untreated patients with high-risk Rai stage 0-II CLL aged 18 years or older	Alemtuzumab + Rituximab	30	-	-
Lenalidomide as Chemoprevention in Treating Patients With High-Risk, Early-Stage B-Cell Chronic Lymphocytic Leukemia [NCT01649791]	Terminated	-	Untreated patients with high-risk Rai-stage 0-II CLL aged 18 years or older	Lenalidomide	8	-	-
Rituximab, Alemtuzumab, and GM-CSF As First-Line Therapy in Treating Patients With Early-Stage Chronic Lymphocytic Leukemia [NCT00562328]	Completed	2	Untreated patients with high-risk Rai-stage 0-II CLL aged 18 years or older	Alemtuzumab + Rituximab + GM-CSF	33	-	-
Ofatumumab for High-Risk Chronic Lymphocytic Leukemia (CLL)/Small Lymphocytic Lymphoma (SLL) [NCT01243190]	Active, not recruiting	2	Untreated patients with high-risk Rai-stage 0-II CLL aged 18 years or older	Ofatumumab	44 (Estimated Enrollment)	-	-
Preemptive Therapy for High Risk Chronic Lymphoid Leukemia Stage A [NCT03766763]	Recruiting	2	Untreated patients with high-risk Binet Stage A CLL aged 18 years or older	Venetoclax	82 (Estimated Enrollment)	-	-

OS: overall survival.

**Table 2:** Clinical trials in CLL.

Trial	Phase	Setting	Interventions	N. of Patients	PFS	OS	MRD
Ibrutinib-Rituximab or Chemoimmunotherapy for Chronic Lymphocytic Leukemia [38]	3	Untreated patients with CLL or SLL subtype of CLL	Ibrutinib-Rituximab	354	3 years: 89.4%	3 years: 98.8%	12 months (78% of patients): 8.3% negative for MRD in PB
			Chemoimmunotherapy (FCR)	175	3 years: 72.9%	3 years: 91.5%	12 months (58.9% of patients): 59.2% negative for MRD in PB
Venetoclax and Obinutuzumab in Patients with CLL and Coexisting Conditions [40]	3	Untreated patients with CLL	Venetoclax + Obinutuzumab	216	24 months: 88.2%	24 months: 91.8%	3 months: 75.5% negative for MRD in PB and 56.9% in BM
			Chlorambucil + Obinutuzumab	216	24 months: 64.1%	24 months: 93.3%	3 months: 35.2% negative for MRD in PB and 17.1% in BM
Ibrutinib Regimens versus Chemoimmunotherapy in Older Patients with Untreated CLL [41]	3	Untreated patients with CLL aged ≥65	Bendamustine + Rituximab	183	24 months: 74%	24 months: 95%	At cycle 9: 8% negative for MRD in BM
			Ibrutinib	182	24 months: 87%	24 months: 90%	At cycle 9: 1% negative for MRD in BM
			Ibrutinib + Rituximab	182	24 months: 88%	24 months: 94%	At cycle 9: 4% negative for MRD in BM
Ibrutinib plus obinutuzumab versus chlorambucil plus obinutuzumab in first-line treatment of chronic lymphocytic leukaemia (iLLUMINATE): a multicentre, randomised, open-label, phase 3 trial [60]	3	Untreated patients with CLL or SLL either aged 65 years or older or younger than 65 years with coexisting conditions	Ibrutinib + Obinutuzumab	113	Median PFS: not reached at median observation time of 31.3 months (Estimated) 30 months: 79%	Median OS: not reached at median observation time of 31.3 months 30 months: 86%	Overall (median f/up was 31.3 months): 20% negative for MRD in BM and 30% in PB
			Chlorambucil + Obinutuzumab	116	Median PFS: 19 months (Estimated) 30 months: 31%	Median OS: not reached at median observation time of 31.3 months 30 months: 85%	Overall (median f/up was 31.3 months): 17% negative for MRD in BM and 20% in PB
Ibrutinib and Venetoclax for First-Line Treatment of CLL [56]	2	Untreated high-risk (at least one of the following: 17p deletion; mutated TP53; 11q deletion; IGHV-unmutated) and older (≥65) patients with CLL	Ibrutinib + Venetoclax	80	(Estimated) 1 year: 98%	(Estimated) 1 year: 99%	After 12 cycles: 61% negative for MRD in BM
Single-agent ibrutinib in treatment-naïve and relapsed/refractory chronic lymphocytic leukemia: a 5-year experience [61]	1b/2	Patients with relapsed or refractory CLL or SLL	Ibrutinib	101	Median PFS: 51 months (Estimated) 5 years: 44%	Median OS: not reached at median observation time of 61.5 months 5 years: 60%	-
		Untreated symptomatic CLL/SLL patients aged 65 or older		31	Median PFS: not reached at median observation time of 61.5 months (Estimated) 5 years: 92%	Median OS: not reached at median observation time of 61.5 months 5 years: 92%	-
Long-term follow-up of the RESONATE phase 3 trial of ibrutinib vs ofatumumab [58]	3	Previously treated patients with CLL or SLL requiring a new therapy and not eligible for purine analog-based therapy	Ibrutinib	195	Median PFS: not reached at median observation time of 44 months 3 years: 59%	Median OS: not reached 3 years: 74%	-
			Ofatumumab [Note: 68% of patients in this arm crossing over to ibrutinib]	196	Median PFS: 8.1 months 3 years: 3%	Median OS: not reached 3 years: 65%	-
Substantial Susceptibility of Chronic Lymphocytic Leukemia to BCL2 Inhibition: Results of a Phase I Study of Navitoclax in Patients With Relapsed or Refractory Disease [62]	1	Relapsed or refractory CLL	Navitoclax	29	Median PFS: 25 months	-	-
Venetoclax in relapsed or refractory chronic lymphocytic leukaemia with 17p deletion: a multicentre, open-label, phase 2 study [63]	2	Patients aged 18 years or older with del(17p) relapsed or refractory CLL	Venetoclax	107	(Estimated) 12 months: 72%	(Estimated) 12 months: 86.7%	-
Venetoclax-Rituximab in Relapsed or Refractory Chronic Lymphocytic Leukemia [64]	3	Patients aged 18 years or older with relapsed or refractory CLL	Venetoclax + Rituximab	194	2 years overall: 84.9% 2 years patients with del(17p): 81.5% 2 years patients without del(17p): 85.9%	2 years overall: 91.9%	At 9 months: 62.4% negative for MRD in PB
			Bendamustine + Rituximab	195	2 years overall: 36.3% 2 years patients with del(17p): 27.8% 2 years patients without del(17p): 41%	2 years overall: 86.6%	At 9 months: 13.3% negative for MRD in PB

SLL: small lymphocytic lymphoma; FCR: fludarabine, cyclophosphamide, rituximab; OS: overall survival; PB: peripheral blood; BM: bone marrow.
